# Perception and awareness of healthcare professionals toward the applications of artificial intelligence in Egyptian healthcare settings

**DOI:** 10.3389/frai.2025.1700493

**Published:** 2026-01-12

**Authors:** Shimaa Azzam, El-Morsy Ahmed El-Morsy, Amira S. A. Said, Nermin Eissa, Doaa Mahmoud Khalil

**Affiliations:** 1 Department of Public Health Technology, Faculty of Applied Health Science Technology, Beni-Suef University, Beni-Suef, Egypt; 2 Department of Public Health and Community Medicine, Faculty of Medicine, Beni-Suef University, Beni-Suef, Egypt; 3 Department of Clinical Pharmacy, College of Pharmacy, Al Ain University, Abu Dhabi, United Arab Emirates; 4 Department of Biomedical Sciences, College of Health Sciences, Abu Dhabi University, Abu Dhabi, United Arab Emirates

**Keywords:** artificial intelligence, attitude, healthcare, knowledge, perception, practice

## Abstract

**Background:**

Healthcare professionals’ awareness and handling of artificial intelligence applications in healthcare enhance patient outcomes and improve processes. This study aimed to evaluate the perception, attitude, knowledge, and practice of healthcare professionals regarding the application of artificial intelligence in Egyptian healthcare settings.

**Method:**

A cross-sectional study in which 367 healthcare professionals responded to an electronic questionnaire.

**Results:**

Out of 367 participants (234 female), radiology and lab test specialty (36.2%) was the predominant. The mean age was 27.03 years; 51.8% of respondents showed positive perception, 68.7% experienced sub-optimal knowledge, 52.9% expressed negative attitudes, and 53.4% demonstrated a low practice level of AI tools. Younger age was significantly associated with positive perception (adjusted odds ratio (AOR) = 0.905, *p* = 0.020) and higher AI practice (AOR = 0.907, *p* = 0.026). University hospital professionals had 61.4% lower odds of optimal knowledge than private hospital professionals (AOR = 0.386, *p* = 0.046). Men had higher odds of both positive attitudes (AOR = 1.844, *p* = 0.010) and high practice level (AOR = 2.92, *p* < 0.001). Pre-bachelor’s holders had lower odds of positive attitudes (AOR = 0.361, *p* = 0.036), as well as physicians compared to nurses and others (AOR = 0.424, *p* = 0.005). Bachelor’s holders showed lower odds of high AI practice (AOR = 0.388, *p* = 0.017).

**Conclusion:**

Despite moderate perception, most professionals have knowledge, attitude, and practice defects. Mainly, younger age and men showed higher engagement, indicating a need for targeted AI training, especially for older and female professionals.

## 1 Introduction

The term artificial intelligence (AI) refers to certain types of tools created by humans that resemble the inherent intelligence in the human mind (Tai, 2020). AI-derived technology has practical applications in different types of industries, including healthcare (Fritsch et al., 2022). AI applications provide benefits through the use of robots, large data, and algorithms (Dharani and Kirupa Krishnan, 2021). There are many uses of AI in healthcare, including increasing the precision of diagnosis, forecasting patient outcomes, and offering a treatment plan designed for each patient (Johnson et al., 2021). AI applications in nursing include robotics, voice assistants, mobile health, sensor-based technology, and scientific decision support (Abdelkareem et al., 2024).

Virtual and physical AI are the two types of AI used in medicine (Fritsch et al., 2022). The virtual component of medicine includes everything from treatment decision guidance that is based on neural networks to applications such as health record systems (Doumat et al., 2022). The physical part focuses on elderly patient care, intelligent prosthetics for people with disabilities, and robots that assist with procedures (Doumat et al., 2022). AI has been assisting physicians in diagnosing patients, determining the disease causes, providing different treatment options, and determining whether a disease is life-threatening (Merhi, 2023).

Available evidences indicate that AI use in the healthcare system is growing, so healthcare professionals need to be familiar with and aware of the principles and ideas of AI (Serbaya et al., 2024). According to studies from 54 nations presented by Sarwar et al. (2019), respondents had positive opinions about AI, and approximately 75% of them expressed a strong desire to use AI as a diagnostic tool to improve patient outcomes connected to pathology work. The acceptance of AI across various clinical specialties was examined by Maskara et al. (2017). They found that while they were aware of the use of AI technology in their field and that a few of their colleagues were using it themselves, doctors were generally supportive of AI but also saw that its use would affect costs and the empathy factor associated with patient counseling. According to a different study (Oh et al., 2019), doctors had highly positive opinions regarding the use of AI in the healthcare system.

Several studies have been done to determine awareness and perceptions of healthcare professionals regarding AI. However, most of these studies have focused on countries with high income rates and developed healthcare systems, often concentrating on specific professional groups or relying mainly on quantitative methods (Sahoo et al., 2025; Lambert et al., 2023; Scott et al., 2021). As a result, they offer a somewhat limited understanding of the challenges and opportunities associated with AI adoption within different healthcare environments. In Egypt, research on AI in healthcare remains scarce, and the available studies focus on physicians or medical students, not considering other important groups such as nurses and technicians (Allam et al., 2024; Issa et al., 2024). Our study aims to address these gaps by providing a condensed analysis of the perception, knowledge, attitude, and practice of a wide range of healthcare professionals in Egypt regarding AI applications. By employing quantitative and qualitative data, this research provides a more specific understanding of how AI is handled in real-world clinical settings. Thus, it provides a unique and timely perspective that can inform national digital health strategies and support the effective integration of AI within the Egyptian healthcare system.

## 2 Materials and methods

### 2.1 Ethical considerations

Ethical approval for conducting this study has been obtained from the Research Ethics Committee, Faculty of Medicine, Beni-Suef University. Approval No: FMBSUREC/03112023/Abdel-Azeem. Participants received a thorough explanation of the study’s goals before participation, and they had the option to share in the research or not; their informed consent was acquired.

### 2.2 Study design

A cross-sectional study through a questionnaire to healthcare workers all over Egypt between January 2024 and June 2024 was conducted.

### 2.3 Population and sampling

Using Epi-info stat-calc, the sample size for the population survey for prevalence of optimal knowledge (>50%) was calculated at 90% confidence level, 5% acceptable margin of error, 1 design effect, and 50% expected frequency (of optimal knowledge). The sample size was found to be 270 healthcare professionals, and the final collected data were obtained from 367 participants covering a wide range of healthcare professionals, including physicians, therapists, pharmacists, and nurses and others (applied health science technologists and technicians). A stratified random sampling strategy was used in the study (Zaman and Bulut, 2023).

### 2.4 Inclusion criteria

Participants must be actively employed in healthcare settings, including men and women of all age groups.

### 2.5 Exclusion criteria

Incomplete filling of questionnaires was excluded from the study.

### 2.6 Data collection

The primary tool for collecting data was a structured questionnaire. A thorough literature review served as the basis for this questionnaire (Serbaya et al., 2024; Oh et al., 2019; Schepman and Rodway, 2020; Sallam et al., 2023; Sommer et al., 2024; Khalf et al., 2022; Mehta et al., 2021). The questionnaire included independent variables (socio-demographics) and dependent variables (perception, knowledge, attitude, and practice) regarding AI applications in healthcare settings. The questionnaire combined a Likert scale and closed-ended questions to make quantitative data collection easier. It consists of the following parts:

#### 2.6.1 Part one: socio-demographic characteristics of the participants

It includes age, sex, qualifications, job, specialty, workplace, years of experience, and residence.

#### 2.6.2 Part two: perception of healthcare professionals regarding the application of AI in healthcare settings

It consists of 47 questions. Questions (1–31) assess familiarity with AI, determine whether AI will replace human jobs in healthcare, measure the impact of AI on clinical efficacy, assess opinion on how AI may restrict roles of healthcare providers in decision-making tasks only, evaluate expectations for AI-derived feedback process between patients and healthcare professionals, determine whether the absence of human in AI-assisted care may affect relationships between patient and healthcare professionals, identify if healthcare professionals of younger age have more motivation to work in technological environment, determine opinions about the concept so that the healthcare professional’s judgment should always be prioritized over AI recommendations, and identify acceptance of use of robots in medical devices transporting, patient mobility aiding, disinfection tasks, and giving greetings to patients and visitors. Questions (32–40) assess perceived benefits of AI in healthcare, such as diagnosis accuracy and patient care process enhancement, human error reduction, disease forecasting, comfortable performance, and rapid access to high-quality clinical data. Questions (41–47) evaluate perceived concerns about AI’s rigidity and limitations in unexpected or controversial cases, inability to adapt to each patient’s preferences, absence of emotions, safety concerns from misleading or underdeveloped algorithms, ethical problems, concerns from the fact that the AI developers are non-medical professionals, and fear of displacement of jobs.

#### 2.6.3 Part three: knowledge of healthcare professionals about the application of AI in healthcare settings

It consists of five questions assessing the participants’ understanding and awareness of AI, especially in the field of healthcare, determining knowledge of the important subfields of AI, assessing awareness of specialized AI tools for diagnostic imaging, and evaluating understanding of AI applications in real-time or long-term patient care.

#### 2.6.4 Part four: attitude of healthcare professionals toward the application of AI in healthcare settings

It consists of five questions assessing the attitude through knowing the views about the effect of AI on the quality of the care process, evaluating the level of acceptance and confidence in AI-based decision-making in clinical settings, identifying beliefs about automation of roles through AI dependance, measuring trust in the safety, accuracy, and reliability of AI tools, and determining whether AI is considered as a solution that minimizes errors in diagnosis, treatment, and data dealings.

#### 2.6.5 Part five: healthcare professionals’ practice toward the application of AI in healthcare settings

It consists of seven questions. It determines the frequency and level of AI integration in the routine clinical activities, determine the extent to which AI is utilized for diagnosis, evaluate AI role in preparing treatment plans to each individualized patient, identify whether AI had been utilized for long term or remote monitoring of patients, assess exposure to educations related to AI, measure perceived effect of AI on work productivity, time management, and task achievement, and capture general user satisfaction, experience, and the perceived AI tools reliability.

### 2.7 Scoring

The answers to perception, attitude, and practice questions have been assessed with a five-point Likert scale (1–5).

For perception and attitude questions, strongly agree = 1 point, agree = 2 points, neutral = 3 points, disagree = 4 points, and strongly disagree = 5 points.

Regarding practice questions (1–5), always = 1 point, often = 2 points, sometimes = 3 points, rarely = 4 points, and never = 5 points. Regarding practice Questions 6 and 7, strongly agree = 1 point, agree = 2 points, neutral = 3 points, disagree = 4 points, and strongly disagree = 5 points.

The answers to knowledge questions have been assessed with a two-point dichotomous scale (true answer = 1 point and wrong/ do not know answers = 0 points).

Participants are categorized according to the median as having either a positive or negative perception or attitude, optimal or sub-optimal knowledge, or high or low practice.

### 2.8 Data collection procedure

An online questionnaire link was raised through the official online groups and digital platforms of participating medical settings. These included their authenticated WhatsApp groups, online forums, and announcement boards. Participation was voluntary, and participants provided informed consent before starting the survey.

A total of 446 clinicians received the survey link, of whom 367 completed the questionnaire (completion rate = 82.3%). As the distribution relied on online organizational groups and voluntary participation, potential sampling biases, including self-selection bias and limited coverage of healthcare professionals not active on digital platforms, are acknowledged in the Limitations section.

### 2.9 Statistical analysis

Statistical analysis was done using the SPSS program, version 25 (SPSS, Chicago, IL, United States), for analysis of the association between socio-demographic characteristics and different score categories. Multi-comparison using the Chi-square test with Bonferroni correction and binary logistic regression was done for univariable and multivariable analysis of the effect of socio-demographic factors on various parameters. The Pearson correlation test was done to determine the correlation between different scores. A significance level of *p* ≤ 0.05 was applied.

## 3 Results

### 3.1 Socio-demographic characteristics

Socio-demographic characteristics are represented in Table 1.

**Table 1:** Socio-demographic characteristics of the studied participants*.

Parameter	Value
Age [Mean (SD), Range]	(27.03 (6.82), 17–61)
Sex [Frequency (%)]	
Male	133 (36.2%)
Female	234 (63.8%)
Residence [Frequency (%)]	
Urban	206 (56.1%)
Rural	161 (43.9%)
Qualification [Frequency (%)]	
Pre-bachelor’s degree	80 (21.8)
Bachelor’s degree	229 (62.4)
Post-bachelor’s degree	58 (15.8)
Job [Frequency (%)]	
Physician	129 (35.1)
Pharmacist	72 (19.6)
Nurse and others	166 (45.2)
Specialty [Frequency (%)]	
Internal medicine and surgery	123 (33.5)
Radiology and lab tests	133 (36.2)
Dentistry	32 (8.7)
Physical therapy	14 (3.8)
Pharmacy	65 (17.7)
Workplace [Frequency (%)]	
Governmental hospital	258 (70.3%)
University hospital	68 (18.5%)
Private hospital	41 (11.2%)
Years of experience (Mean (SD), Median, Interquartile Range)	(4.42 (5.66), 3, 4)

*Values are presented as the frequency (%) of a total of 367 participants. SD, Standard Deviation.

### 3.2 The results of the survey responses

The total responses regarding the perception, knowledge, attitude, and practice of healthcare professionals regarding AI application in healthcare settings are shown in the Supplementary Tables 1–4, respectively.

### 3.3 Scoring

Descriptive analysis of scores of various assessment points {perception, practice, attitude [standardized on the Likert scale (1–5)], and knowledge [based on the number of questions (0–7)]} and score categories based on the median (*n* = 367) are represented in Table 2, and a chart showing the various score categories’ frequencies is represented in Supplementary Figure 1.

**Table 2:** Assessment points scores and a median-based score categories.

Score descriptives	Mean (SD)	Median	Range	Score categories	Frequency (%)
Perception	3.27 (0.32)	3.26	1.94–4.19	Positive perception	190 (51.8)
				Negative perception	177 (48.2)
Knowledge	4.66 (1.35)	5	1–7	Optimal Knowledge	115 (31.3)
				Sub-optimal Knowledge	252 (68.7)
Attitude	3.29 (0.73)	3.2	1–5	Positive attitude	173 (47.1)
				Negative attitude	194 (52.9)
Practice	2.63 (0.91)	2.57	1–5	High practice	171 (46.6)
				Low practice	196 (53.4)

SD, Standard deviation.

### 3.4 Association between socio-demographic characteristics and different score categories

Multi-comparison using the Chi-square test with Bonferroni correction, followed by binary logistic regression for univariable and multivariable analysis of socio-demographic characteristics associated with positive perception, optimal knowledge, positive attitude, and high practice, is represented in Tables 3–6.

**Table 3:** Factors affecting healthcare professionals’ perception regarding the application of AI in healthcare settings.

Socio-demographic characteristics	Perception categories		Unadjusted OR (95% CI)	*p*-value	AOR (95% CI)	*p*-value
	Positive perception	Negative perception				
Age [Mean (SD)]	26.59 (7.24)	27.51 (6.34)	0.980 (0.951–1.011)	0.200	0.905 (0.832–0.984)	0.020**
Sex [Frequency (%)]						
Male	71 (53.4) ^a^	62 (46.6) ^a^	1.107 (0.723–1.695)	0.641	1.314 (0.823–2.098)	0.252
Female	119 (50.9) ^a^	115 (49.1) ^a^	Reference category			
Qualification [Frequency (%)]						
Pre-bachelor’s degree	48 (60) ^a^	32 (40) ^a^	1.306 (0.660–2.586)	0.443	0.703 (0.276–1.792)	0.461
Bachelor’s degree	111 (48.5) ^a^	118 (51.5) ^a^	0.819 (0.460–1.459)	0.499	0.641 (0.308–1.335)	0.235
Post-bachelor’s degree	31 (53.4) ^a^	27 (46.6) ^a^	Reference category			
Job [Frequency (%)]						
Physician	66 (52.4) ^a^	63 (47.6) ^a^	0.764 (0.481–1.213)	0.254	1.035 (0.578–1.853)	0.908
Pharmacist	28 (38.9) ^b^	44 (61.1) ^a^	0.464 (0.264–0.817)	0.008*	2.226 (0.383–12.930)	0.373
Nurse and others	96 (57.8) ^b^	70 (42.2) ^a^	Reference category			
Specialty [Frequency (%)]						
Internal medicine and surgery	70 (56.9) ^a^	53 (43.1) ^a^	2.412 (1.296–4.489)	0.005*	4.688 (0.762–28.847)	0.096
Radiology and laboratory tests	76 (57.1) ^a^	57 (42.9) ^a^	2.435 (1.318–4.497)	0.004*	4.852 (0.789–29.838)	0.088
Dentistry	13 (40.6) ^a^	19 (59.4) ^a^	1.249 (0.524–2.981)	0.616	2.721 (0.375–19.728)	0.322
Physical therapy	8 (57.1) ^a^	6 (42.9) ^a^	2.435 (0.752–7.878)	0.137	5.540 (0.628–48.862)	0.123
Pharmacy	23 (35.4) ^b^	42 (64.6) ^a^	Reference category			
Workplace [Frequency (%)]						
Governmental hospital	141 (54.7) ^a^	117 (45.3) ^a^	1.883 (0.960–3.693)	0.066	1.926 (0.933–3.976)	0.076
University hospital	33 (48.5) ^a^	35 (51.5) ^a^	1.473 (0.670–3.237)	0.335	1.781 (0.748–4.240)	0.192
Private hospital	16 (39) ^a^	25 (61) ^a^	Reference category			
Years of experience [Mean (SD), Median (IQR)]	4.47 (5.9), 3 (3)	4.36 (5.39), 3 (4)	1.004 (0.968–1.041)	0.842	1.093 (0.998–1.198)	0.056
Residence [Frequency (%)]						
Urban	111 (53.9) ^a^	95 (46.1) ^a^	1.213 (0.803–1.833)	0.360	1.420 (0.902–2.238)	0.130
Rural	79 (49.1) ^a^	82 (50.1) ^a^	Reference category			

Values in the same row and sub-table not sharing the same subscript are significantly different at p < 0.05 in the two-sided test of equality for column proportions. *: Significantly different unadjusted odds with reference category (*p*<0.05). **Significant effect on perception based on multivariable analysis (*p*<0.05). AOR, Adjusted Odds Ratio; CI, Confidence Interval; IQR, Inter Quartile Range; OR, Odds Ratio; SD, Standard Deviation.

**Table 4:** Factors affecting healthcare professionals’ knowledge about the application of AI in healthcare settings.

Socio-demographic characteristics	Knowledge categories		Unadjusted OR (95% CI)	*p*-value	AOR (95% CI)	*p*-value
	Optimal knowledge	Sub-optimal knowledge				
Age [Mean (SD)]	27.25 (7.1)	26.93 (6.71)	1.007 (0.975–1.039)	0.677	1.007 (0.927–1.093)	0.870
Sex [Frequency (%)]						
Male	48 (36.1) ^a^	85 (63.9) ^a^	1.408 (0.894–2.215)	0.139	1.380 (0.850–2.240)	0.193
Female	67 (28.6) ^a^	167 (71.4) ^a^	Reference category			
Qualification [Frequency (%)]						
Pre-bachelor’s degree	22 (27.5) ^a^	58 (72.5) ^a^	0.668 (0.323–1.382)	0.277	0.412 (0.154–1.100)	0.077
Bachelor’s degree	72 (31.4) ^a^	157 (68.6) ^a^	0.808 (0.442–1.478)	0.489	0.558 (0.259–1.202)	0.136
Post-bachelor’s degree	21 (36.2) ^a^	37 (63.8) ^a^	Reference category			
Job [Frequency (%)]						
Physician	43 (33.3) ^a^	86 (66.7) ^a^	1.096 (0.671–1.792)	0.714	0.919 (0.496–1.703)	0.788
Pharmacist	20 (27.8) ^a^	52 (72.2) ^a^	0.843 (0.458–1.554)	0.584	0.655 (0.116–3.712)	0.633
Nurse and others	52 (31.3) ^a^	114 (68.7) ^a^	Reference category			
Specialty [Frequency (%)]						
Internal medicine and surgery	44 (35.8) ^a^	79 (64.2) ^a^	1.454 (0.754–2.804)	0.264	1.051 (0.175–6.330)	0.957
Radiology and laboratory tests	36 (27.1) ^a^	97 (72.9) ^a^	0.969 (0.499–1.883)	0.926	0.695 (0.114–4.236)	0.694
Dentistry	11 (34.4) ^a^	21 (65.6) ^a^	1.368 (0.551–3.396)	0.500	1.015 (0.140–7.377)	0.989
Physical therapy	6 (42.9) ^a^	8 (57.1) ^a^	1.958 (0.596–6.436)	0.268	1.243 (0.145–10.672)	0.843
Pharmacy	18 (27.7) ^a^	47 (72.3) ^a^	Reference category			
Workplace [Frequency (%)]						
Governmental hospital	86 (33.3) ^a^	172 (66.7) ^a^	0.867 (0.436–1.721)	0.683	0.946 (0.458–1.952)	0.880
University hospital	14 (20.6) ^b^	54 (79.4) ^a^	0.449 (0.189–1.068)	0.070	0.386 (0.152–0.984)	0.046**
Private hospital	15 (36.6) ^a^	26 (63.4) ^a^	Reference category			
Years of experience [Mean (SD), Median (IQR)]	4.54 (6.25), 3 (3)	4.36 (5.38), 3 (4)	1.006 (0.968–1.045)	0.779	0.986 (0.900–1.080)	0.761
Residence [Frequency (%)]						
Urban	61 (29.6) ^a^	145 (70.4) ^a^	0.834 (0.535–1.299)	0.421	0.760 (0.470–1.227)	0.261
Rural	54 (33.5) ^a^	107 (66.5) ^a^	Reference category			

Values in the same row and sub-table not sharing the same subscript are significantly different at *p* < 0.05 in the two-sided test of equality for column proportions. **Significant effect on knowledge based on multivariable analysis (*p* <0.05).AOR: Adjusted Odds Ratio. CI, Confidence Interval; IQR, Inter Quartile Range; OR, Odds Ratio; SD, Standard Deviation.

**Table 5:** Factors affecting healthcare professionals’ attitude toward the application of AI in healthcare settings.

Socio-demographic characteristics	Attitude categories		Unadjusted OR (95%CI)	*p*-value	AOR (95%CI)	*p*-value
	Positive attitude	Negative attitude				
Age [Mean (SD)]	27.12 (6.81)	26.95 (6.86)	1.004 (0.974–1.034)	0.814	0.972 (0.897–1.052)	0.481
Sex [Frequency (%)]						
Male	74 (55.6) ^b^	59 (44.4) ^a^	1.710 (1.113–2.627)	0.014*	1.844 (1.154–2.947)	0.010**
Female	99 (42.3) ^b^	135 (57.7) ^a^	Reference category			
Qualification [Frequency (%)]						
Pre-bachelor’s degree	37 (46.3) ^a^	43 (53.7) ^a^	0.749 (0.381–1.476)	0.404	0.361 (0.139–0.936)	0.036**
Bachelor’s degree	105 (45.9) ^a^	124 (54.1) ^a^	0.738 (0.414–1.314)	0.302	0.525 (0.248–1.113)	0.093
Post-bachelor’s degree	31 (53.4) ^a^	27 (46.4) ^a^	Reference category			
Job [Frequency (%)]						
Physician	49 (38) ^b^	80 (62) ^a^	0.493 (0.308–0.788)	0.003*	0.424 (0.233–0.770)	0.005**
Pharmacist	32 (44.4) ^a^	40 (55.6) ^a^	0.643 (0.369–1.123)	0.121	1.443 (0.259–8.032)	0.675
Nurse and others	92 (55.4) ^b^	74 (44.6) ^a^	Reference category			
Specialty [Frequency (%)]						
Internal medicine and surgery	62 (50.4) ^a^	61 (49.6) ^a^	1.430 (0.780–2.624)	0.248	3.548 (0.603–20.885)	0.161
Radiology and laboratory tests	68 (51.1) ^a^	65 (48.9) ^a^	1.472 (0.809–2.680)	0.206	3.792 (0.648–22.199)	0.139
Dentistry	13 (40.6) ^a^	19 (59.4) ^a^	0.963 (0.407–2.278)	0.932	3.626 (0.515–25.523)	0.196
Physical therapy	3 (21.4) ^b^	11 (78.6) ^a^	0.384 (0.098–1.508)	0.170	1.219 (0.128–11.583)	0.863
Pharmacy	27 (41.5) ^a^	38 (58.5) ^a^	Reference category			
Workplace [Frequency (%)]						
Governmental hospital	126 (48.8) ^a^	132 (51.2) ^a^	1.105 (0.571–2.140)	0.767	1.003 (0.488–2.064)	0.993
University hospital	28 (41.2) ^a^	40 (58.8) ^a^	0.811 (0.371–1.770)	0.598	0.710 (0.298–1.693)	0.440
Private hospital	19 (46.3) ^a^	22 (53.7) ^a^	Reference category			
Years of experience [Mean (SD), Median (IQR)]	4.73 (5.39), 3 (3)	4.14 (5.88), 2 (3)	1.019 (0.982–1.057)	0.321	1.031 (0.945–1.125)	0.495
Residence [Frequency (%)]						
Urban	103 (50) ^a^	103 (50) ^a^	1.300 (0.859–1.967)	0.215	1.407 (0.890–2.223)	0.144
Rural	70 (43.5) ^a^	91 (56.5) ^a^	Reference category			

Values in the same row and sub-table not sharing the same subscript are significantly different at *p* < 0.05 in the two-sided test of equality for column proportions. *Significantly different unadjusted odds with reference category (*p*< 0.05). **Significant effect on perception based on multivariable analysis (*p* <0.05). AOR, Adjusted Odds Ratio; CI, Confidence Interval; IQR, Inter Quartile Range; OR, Odds Ratio; SD, Standard Deviation.

**Table 6:** Factors affecting the practice level of AI applications among healthcare professionals in healthcare settings.

Socio-demographic characteristics	Practice categories		Unadjusted OR (95% CI)	*p*-value	AOR (95% CI)	*p*-value
	High practice	Low practice				
Age [Mean (SD)]	25.74 (5.4)	28.16 (7.7)	0.942 (0.909–0.977)	0.001*	0.907 (0.833–0.988)	0.026**
Sex [Frequency (%)]						
Male	79 (59.4) ^b^	54 (40.6) ^a^	2.258 (1.463–3.486)	<0.001*	2.920 (1.786–4.772)	<0.001**
Female	92 (39.3) ^b^	142 (60.7) ^a^	Reference category			
Qualification [Frequency (%)]						
Pre-bachelor’s degree	43 (53.75) ^a^	37 (46.25) ^a^	1.245 (0.633–2.451)	0.526	0.485 (0.182–1.293)	0.148
Bachelor’s degree	100 (43.7) ^a^	129 (56.3) ^a^	0.831 (0.466–1.480)	0.529	0.388 (0.179–0.842)	0.017**
Post-bachelor’s degree	28 (48.3) ^a^	30 (51.7) ^a^	Reference category			
Job [Frequency (%)]						
Physician	56 (43.4) ^a^	73 (56.6) ^a^	0.767 (0.483–1.218)	0.261	0.826 (0.453–1.508)	0.535
Pharmacist	32 (44.4) ^a^	40 (55.6) ^a^	0.800 (0.459–1.394)	0.431	1.356 (0.268–6.854)	0.713
Nurse and others	83 (50) ^a^	83 (50) ^a^	Reference category			
Specialty [Frequency (%)]						
Internal medicine and surgery	61 (49.6) ^a^	62 (50.4) ^a^	1.300 (0.710–2.381)	0.395	1.890 (0.349–10.232)	0.460
Radiology and laboratory tests	59 (44.4) ^a^	74 (55.6) ^a^	1.054 (0.579–1.917)	0.864	1.753 (0.324–9.465)	0.514
Dentistry	14 (43.75) ^a^	18 (56.25) ^a^	1.028 (0.438–2.414)	0.950	1.729 (0.265–11.284)	0.567
Physical therapy	9 (64.3) ^a^	5 (35.7) ^a^	2.379 (0.718–7.884)	0.156	4.810 (0.583–39.672)	0.145
Pharmacy	28 (43.1) ^a^	37 (56.9) ^a^	Reference category			
Workplace [Frequency (%)]						
Governmental hospital	116 (45) ^a^	142 (55) ^a^	0.778 (0.402–1.505)	0.456	1.067 (0.510–2.231)	0.864
University hospital	34 (50) ^a^	34 (50) ^a^	0.952 (0.439–2.068)	0.902	1.563 (0.644–3.793)	0.324
Private hospital	21 (51.2) ^a^	20 (48.8) ^a^	Reference category			
Years of experience [Mean (SD), Median (IQR)]	5.16 (6.83), 3 (4)	3.57 (3.75), 2 (3)	0.945 (0.906–0.987)	0.010*	1.003 (0.912–1.104)	0.943
Residence [Frequency (%)]						
Urban	99 (48.1) ^a^	107 (51.9) ^a^	1.144 (0.756–1.730)	0.525	1.263 (0.797–2.001)	0.321
Rural	72 (44.7) ^a^	89 (55.3) ^a^	Reference category			

Values in the same row and sub-table not sharing the same subscript are significantly different at *p* < 0.05 in the two-sided test of equality for column proportions. *Significantly different unadjusted odds with reference category (*p*< 0.05). **Significant effect on perception based on multivariable analysis (*p* <0.05). AOR, Adjusted Odds Ratio; CI, Confidence Interval; IQR; Inter Quartile Range; OR, Odds Ratio; SD, Standard Deviation.

Table 3 shows that younger age was significantly associated with a more positive perception, with an adjusted odds ratio (AOR) of 0.905 (95% confidence interval (CI): 0.832–0.984; *p* = 0.020).

Table 4 shows that only the workplace was a statistically significant determinant of optimal knowledge about AI applications in healthcare after adjustment (*p* < 0.05). Healthcare professionals working in university hospitals had 61.4% lower odds of optimal knowledge of AI compared to those in private hospitals, AOR = 0.386 (95% CI: 0.152–0.984, *p* = 0.046).

Table 5 shows that sex, qualifications, and job had a statistically significant effect on determining the attitude toward AI application in healthcare settings. Male healthcare professionals had 1.84 times higher odds of positive attitudes compared to female healthcare professionals, AOR = 1.844 (95% CI: 1.154–2.947, *p* = 0.010). Professionals with pre-bachelor’s degrees showed 64% lower odds of positive attitudes against post-bachelor’s holders, AOR = 0.361 (95% CI: 0.139–0.936, *p* = 0.036). Physicians demonstrated 58% lower odds of positive attitudes compared to nurses and others, AOR = 0.424 (95% CI: 0.233–0.770, *p* = 0.005).

Table 6 shows that age, sex, and qualification level had a statistically significant effect on the high practice level of AI in healthcare settings. Younger participants were significantly more likely to report high levels of practice, with a mean age of 25.74 years compared to 28.16 years among those with low practice, AOR = 0.907 (95% CI: 0.833–0.988, *p* = 0.026), Men showed 2.9 times higher odds of high practice than women, AOR = 2.92 (95% CI: 1.786–4.772, *p* < 0.001). Bachelor’s degree holders had 61% lower odds of high practice against post-bachelor’s, AOR = 0.388 (95% CI: 0.179–0.842, *p* = 0.017).

### 3.5 Scores correlation

There was a significant correlation between attitude score and other scores (*p*-value < 0.01). Moreover, practice score had a significant correlation with perception score at *p*-value <0.01. In contrast, it had a significant correlation with knowledge score at *p*-value <0.05. On the other hand, there was no significant correlation between knowledge and perception score (*p*-value = 0.068; Table 7).

**Table 7:** Correlation between different scores.

Score		Pearson correlation	*p*-value (2-tailed)
Attitude	Practice	0.610**	< 0.001
	Knowledge	0.140**	0.007
	Perception	0.541**	< 0.001
Practice	Knowledge	0.121*	0.021
	Perception	0.400**	< 0.001
Knowledge	Perception	0.095	0.068

**Correlation is significant at the 0.01 level (2-tailed). *Correlation is significant at the 0.05 level (2-tailed).

## 4 Discussion

The use of AI tools in healthcare requires professionals’ awareness and good handling of these tools, which influence patients’ outcomes, process development, and good AI integration (Catalina et al., 2023; Gazquez-Garcia et al., 2025). In this study, 367 healthcare professionals participated in providing their responses regarding an online questionnaire aiming to assess their perception, attitude, knowledge, and practice regarding the applications of AI in Egyptian healthcare settings to help decision makers put their hands on opinions, familiarity, and concerns for the best integration of AI in healthcare.

This study showed that the mean (SD) perception score value was 3.27 (0.32). Participants were categorized as having positive or negative perceptions based on the median score of 3.26, considering a total score of 5. There were 51.8% of participants who had a positive perception of the application of AI in healthcare settings. These indicate that slightly more than half of the participants have an optimistic look toward AI applications in healthcare settings. In contrast, nearly the other half may have concerns that should be addressed. These concerns may be worries about losing the job, ethical challenges, or privacy concerns related to the use of AI. This is consistent with the results of Sabra et al. (2023), who reported that “the majority of participants had a moderate perception toward the application of AI in health care” (Sabra et al., 2023). This balance shows the value of focused instruction and involvement to build confidence and prepare for AI integration. The present study showed that age is a significant determinant of positive perception toward AI applications in healthcare settings, with younger participants showing a more positive perception (AOR 0.905, *p* = 0.020). This aligns with previous research, which found that younger individuals may be more receptive to new technologies; this may be due to adaptability or recent educational methods (Volkom et al., 2014; Czaja et al., 2006). The lack of significant associations for other variables indicates that socio-demographic characteristics may have a limited effect beyond age in determining perception. Therefore, it is essential to target younger professionals when introducing new technologies; however, specified strategies should be considered to engage older professionals effectively.

The mean (SD) knowledge score value was 4.66 (1.35). Participants were categorized as having optimal or sub-optimal knowledge based on the median score of 5, considering a total score of 7. There were 68.7% of participants who had sub-optimal knowledge about the application of AI in healthcare settings. These results are similar to those found by Abdullah and Fakieh (2020), where there was a lack of overall knowledge about artificial intelligence (Abdullah and Fakieh, 2020). This represents a gap in knowledge, which may prevent the adoption and use of AI technologies in clinical practice effectively. This shows the importance of targeted education to enhance awareness among healthcare professionals.

Regarding factors associated with optimal knowledge, the significantly lower AI knowledge among university hospital professionals (AOR = 0.386, *p* = 0.046) is considered against expectations, as academic hospitals are a good place for thinking and awareness. However, this can be explained by the fact that university hospital staff may consider clinical and teaching roles as a priority over AI education (Ali, 2025). The lack of significant association between higher qualifications (e.g., post-bachelor’s) and AI knowledge indicates that formal education alone may not ensure AI knowledge. This shows the need for targeted AI training programs beyond traditional curricula.

The mean (SD) attitude score value was 3.29 (0.73). Participants were classified as having positive or negative attitudes based on the median score of 3.2, considering a total score of 5. There were 52.9% of participants who had a negative attitude toward the application of AI in healthcare settings. This indicates a common worry among medical practitioners that might prevent AI technology from being integrated. Addressing these fears and misconceptions through training and transparent communication is essential to changing attitudes positively. This is in contrast to Sabra et al. (2023) and Abdelkareem et al. (2024), who found that “more than 64% of participants had a positive attitude toward the application of AI in health care” (Abdelkareem et al., 2024; Sabra et al., 2023). This study revealed three significant predictors of positive attitudes toward AI in healthcare. Male healthcare professionals are more receptive to AI (AOR = 1.844, *p* = 0.010), aligning with global trends, showing an increased technology acceptance rate among men (Liu and Guo, 2017). This may reflect persistent sex gaps in Science, Technology, Engineering, and Mathematics (STEM) confidence or workplace dynamics that influence technological attitude (Wang and Degol, 2017). The second predictor is qualification level, while pre-bachelor’s holders showed significantly lower attitude, the non-significance of bachelor’s degrees (*p* = 0.093) indicates a borderline effect where only post-bachelor’s degree positively affects attitude. This shows that higher educational levels improve attitude toward AI technologies (Sharma and Singh, 2024). The third predictor is the healthcare professional’s job. Physicians’ lower attitude compared to nurses is in contrast with studies showing physicians’ leading position in AI adoption (Ehrenfeld, 2023). Explanations include physicians’ concerns about diagnosis independence and increased exposure of nurses to AI-powered workflow tools (Gentil et al., 2025).

The mean (SD) practice score value was 2.63 (0.91). Participants were categorized into high and low practice based on the median score of 2.57, considering a total score of 5. There were 53.4% of the participants who experienced a low level of AI practice in healthcare settings. This represents a gap between awareness and actual implementation of AI technologies, indicating the need for more practical training programs and institutional support to translate knowledge and attitudes into effective practice. Nasr and his colleagues in 2025 reported that “29% of participants stated that they use AI at least once a week,” which represents a low practice consistent with our findings (Nasr et al., 2025). Moreover, Swed et al. (2022) reported that “89.3% of healthcare professionals had never applied AI in their work” (Swed et al., 2022). Our study found a significant association between high practice of AI applications and younger age, where the AOR = 0.907 (*p* = 0.026), indicating that each additional year of age was associated with a 9.3% reduction in the odds of high practice. Moreover, men showed 2.9 times higher odds of high practice than women, where the AOR = 2.92 (*p* < 0.001). This may be explained by the fact that younger and male healthcare professionals may be more actively engaged with AI tools, possibly due to greater exposure or confidence in using technology (Nesar et al., 2023).

Regarding the correlation between scores, attitude scores were significantly correlated with other scores (*p* < 0.001), indicating a strong interrelationship between participants’ attitudes and their overall engagement with AI. Rony et al. (2024) reported that optimum knowledge is associated with a positive attitude toward AI application in healthcare (Rony et al., 2024). Additionally, practice scores showed significant correlations with both perception (p < 0.001) and knowledge scores (*p* < 0.05). This is similar to the results obtained by Rony et al. (2024) and Alabbad et al. (2025), who reported a significant positive correlation between knowledge score and each attitude and practice score, and also between attitude and practice scores (Rony et al., 2024; Alabbad et al., 2025). On the other hand, no significant correlation was found between knowledge and perception scores (*p* = 0.068), which indicates that awareness alone may not directly influence perception without practical exposure or attitudinal alignment.

Healthcare information technology acceptance is strongly impacted by effort expectancy, performance expectancy, and attitudes toward use, as defined by the Technology Acceptance Model (TAM) and Unified Theory of Acceptance and Use of Technology (UTAUT), according to a thorough meta-analysis synthesizing 214 independent samples (more than 83,000 users). The study emphasizes the need for context-specific measures to enhance adoption by highlighting how acceptance differs by technology type, user demographics, and healthcare setting (Chong et al., 2022). Performance expectations and enabling circumstances are the most reliable adoption drivers, according to a recent scoping review that mapped 46 papers on clinician acceptance and usage of AI in healthcare. However, obstacles such as physician opposition, ethical and legal issues, and technical aspects such as design quality all have a significant impact. The review emphasizes the necessity of improving current frameworks to better capture context-specific causes and fill in gaps, particularly in low- and middle-income nations (Scipion et al., 2025).

To reduce the gap for using AI tools and to increase awareness and knowledge about AI in healthcare professionals, the following actions are required: Integration of AI education, such as basic concepts of artificial intelligence, data science, and digital health, in undergraduate and postgraduate healthcare programs to prepare professionals for AI-powered practice; Promotion of collaborative efforts among computer scientists, engineers, and healthcare professionals to narrow the gap between clinical application and technical advancement; Facilitation of the accessibility of digital platforms and AI-based tools in healthcare settings so that professionals can obtain practical experience; Organization of workshops and courses for training on applications of AI in diagnosis, treatment strategies, and healthcare management; Organization of conferences and awareness campaigns that emphasize the advantages, drawbacks, and practical applications of AI in healthcare; Establishment of national frameworks and regulations that assist the government and healthcare organizations in advancing the ethical and safe application of AI technologies.

## 5 Limitations

Study limitations include the use of self-reported data, which may cause bias in response, as participants may have over- or underestimated their knowledge about or use of AI. Moreover, as the survey distribution relied on online organizational groups and voluntary participation, potential sampling biases, including self-selection bias and limited coverage of healthcare professionals, are not active on digital platforms. Moreover, the study sample was limited to a specific geographic and institutional region, which may affect the generalizability of findings to other regions or healthcare systems. Finally, the cross-sectional design was also a limitation of this study. Future studies should include more diverse samples and objective measures of AI handling to validate these findings.

## 6 Conclusion

This study revealed that while just over half of the studied healthcare professionals had a positive perception of AI, most showed sub-optimal knowledge, negative attitudes, and low practice levels. Higher engagement with AI tools was significantly predicted by younger age and male sex. Defects in awareness and practice show the importance of targeted educational and practical training programs. Special consideration should be given to older and female professionals to ensure confident and effective integration of AI technologies within different healthcare settings.

## Data Availability

The raw data supporting the conclusions of this article will be made available by the authors, without undue reservation.
